# Electrocardiogram of a patient with mushroom poisoning‐induced myocarditis

**DOI:** 10.1111/anec.13011

**Published:** 2022-10-20

**Authors:** Zuoan Qin, Li Luo, Liangqing Ge

**Affiliations:** ^1^ Department of Cardiology The First People's Hospital of Changde City Changde China

**Keywords:** atrioventricular block, mushroom poisoning, myocarditis, QT interval prolongation

## Abstract

A patient presented to our hospital with myocarditis caused by mushroom poisoning. The early ECG changes in this patient were very similar to the ECG of hyperacute ST‐segment elevation myocardial infarction or hyperkalemia, but further tests eliminated these options. The patient was fully treated by timely hemodialysis treatment, confirming the diagnosis of mushroom poisoning‐induced myocarditis. Although not specific to mushroom poisoning myocarditis, our experience shows that the observed ECG changes. Our findings have the potential to help diagnose and manage this potentially fatal disease in the future.

## Graphical abstract

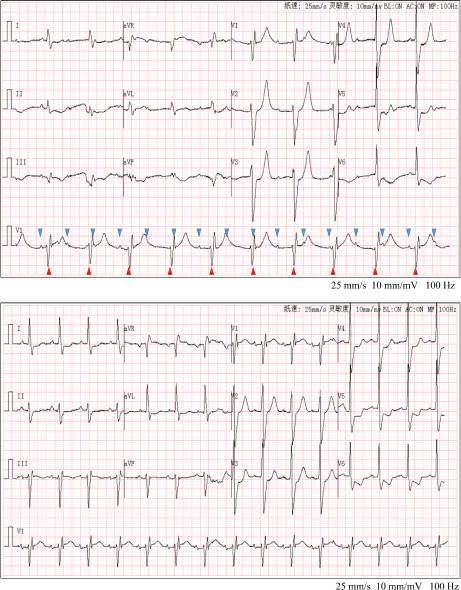

## 1 INTRODUCTION

Myocarditis due to mushroom poisoning is mainly due to myocardial damage caused by the toxins in mushrooms entering the body through the gastrointestinal tract. Poisoning due to the consumption of poisonous mushrooms has become a common clinical problem in China during the summer mushroom growing season. Patients with severe mushroom poisoning may die due to liver and kidney failure, while myocardial damage caused by mushroom poisoning is rare (White et al., 2019). It has been reported that mushroom poisoning can lead to electrocardiogram (ECG) ST‐T changes (ST elevation or depression, T wave inversion), AV block, QT interval prolongation, and a small number of patients also have transient Q waves (Erenler et al., 2016).

## 2 CASE REPORT

**FIGURE 1:**
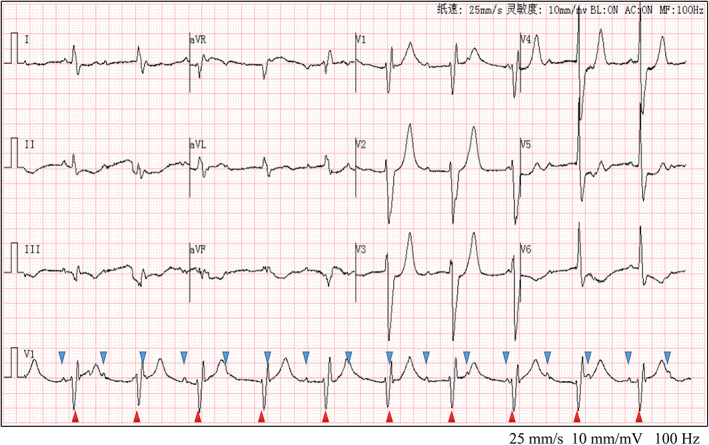
On admission electrocardiogram. The patient's ECG revealed a third‐degree atrioventricular block, Q wave in lead III, complete right bundle branch block, low voltage in limb leads, and QT/QTc: 508/521.

A 55‐year‐old male patient presented to the emergency department with a 2‐day history of dizziness, nausea, and vomiting with dyspnea following the consumption of mushrooms 2 days ago. The main diagnosis was mushroom poisoning. After hemofiltration and anti‐shock treatment, the patient's symptoms improved. Vital signs showed body temperature 36.6°C, heart rate 61 beats/min, respiration rate 24 breaths/min, blood pressure 115/65 mmHg, and oxygen saturation 90%. The ECG of the patient admitted to the emergency department is shown in Figure 1. The P wave and QRS complex in lead V_1_ support the diagnosis of third‐degree atrioventricular (AV) block with accelerated junctional escape rhythm. The anterior leads V_1_–V_4_ indicated a high‐pointed T wave. A Q wave was detected in lead III. In addition, the ECG showed a complete right bundle branch block, low voltage in limb leads, and QT/QTc: 508/521, suggesting prolongation of QT interval. Laboratory test results (normal range in parentheses) showed elevated troponin I at 1.05 (0–0.023) ng/ml, lactate dehydrogenase 737 (120–250) U/L, creatine kinase 403 (50–310) U/L, creatine kinase isoenzyme 93.1 (0–25) U/L, myoglobin 175.4 (50–310) ng/ml, alanine aminotransferase 356 (9–50) U/L, aspartate aminotransferase 758 (15–45) U/L, and creatinine 176 (57–97) μmol/L. Electrolyte levels were normal.

**FIGURE 2:**
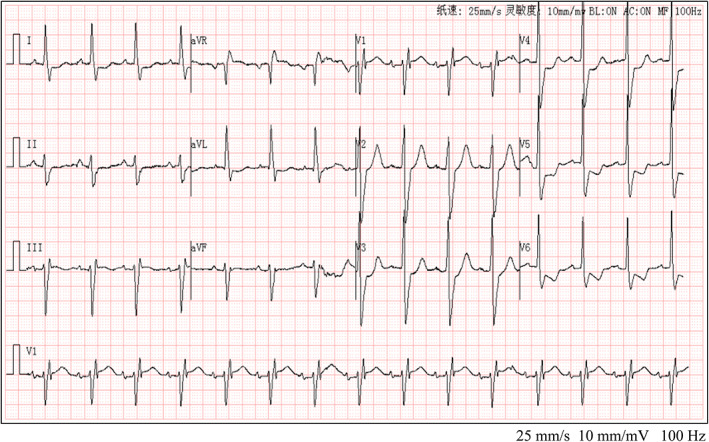
Electrocardiogram of the patient after treatment. Repeat ECG following treatment showed gradual improvement of low voltage and a high sharp T wave, QT/QTc: 402/484, the disappearance of AV block and Q wave, and sinus rhythm.

Preliminary diagnosis included: (1) multiple organ dysfunction syndrome; (2) poisoning by ingestion of mushroom; (3) possible viral myocarditis; (4) possible anterior myocardial infarction (AMI); (5) third‐degree AV block. Cardiac MRI showed myocardial and subendocardial delayed enhancement in the inferior septal wall, inferior wall, and inferior lateral wall of the central segment from the bottom of the heart to the central segment; the left heart was enlarged, and the ventricular wall motion was weakened. Coronary angiography showed normal coronary arteries. Echocardiography results were as follows: the thickness of the left ventricle, 55 mm; left atrium, 33 mm; interventricular septum, 11 mm; left ventricular posterior wall, 12 mm; aortic inner diameter, 31 mm; pulmonary artery inner diameter, 21 mm; and inner diameter of the inferior vena cava, 16 mm; left ventricular ejection fraction, 45%; and mild mitral valve, tricuspid valve, and aortic valve regurgitation. On the 4th day, the patient's ECG showed a disappearance of the third‐degree AV block, a disappearance of the Q wave in lead III, a disappearance of the high‐pointed T wave in the anterior lead, and a shortening of the QRS width and QT interval (Figure 2). Myocardial remodeling, anticoagulation, and liver and kidney protection were improved, and symptomatic treatment was given. The condition improved, and the patient was discharged. The primary diagnosis was acute myocarditis due to mushroom poisoning.

## 3 DISCUSSION

The early ECG changes in this patient are very similar to the ECG of hyperacute ST‐segment elevation myocardial infarction (STEMI) or hyperkalemia. Due to the normal serum potassium at the time of admission, hyperkalemia was excluded. The general elevation of troponin I (cTnI) and myocardial enzymes at the time of admission suggested STMEI; however, unlike typical STEMI, this patient's ECG suggested anterior myocardial infarction (AMI), and yet the patient's arrhythmia was a third‐degree AV block. Clinically, inferior myocardial infarction combined with AV block is more common, while AMI is often combined with ventricular arrhythmia. AMI was finally ruled out by coronary angiography.

Myocarditis due to mushroom toxins is believed to mainly be caused by the poison directly invading the myocardium or causing coronary artery spasms, leading to myocardial ischemia, hypoxia, edema, and metabolic disorders, resulting in the dissolution, necrosis, and apoptosis of myocardial cells. Most of these effects are reversible if the inflammation is controlled and the myocardial blood supply is improved (Machado et al., 2014; Tepetam et al., 2016). On admission, the patient had low voltage in the limb leads, the myocardial damage involved the cardiac conduction system, and the patient developed a third‐degree AV block, indicating a very serious condition. High‐grade AV block is associated with higher mortality (Ogunbayo et al., 2019). The patient's ECG on admission also showed QT prolongation, suggesting that poison‐induced myocarditis caused the negative ventricular delay. However, after treatment, the low voltage of limb leads and long QT interval gradually improved, and the AV block and Q wave disappeared (Figure 2).

Contrary to viral myocarditis, mushroom poison‐induced myocarditis emphasizes the removal and antagonism of harmful toxins (Wennig et al., 2020). As the patient's ECG showed significant improvement on the 4th day, likely due to timely hemodialysis treatment; it confirmed toxic myocarditis rather than viral myocarditis.

## 4 CONCLUSIONS

Mushroom poison‐induced myocarditis can cause uneven repolarization, manifesting as a prolongation of the QT interval. Extensive myocardial cell damage can cause low voltage in limb leads and ST changes in multiple leads. If the myocardial conduction system is involved, it can also lead to AV block performance. Although not specific to mushroom poison‐induced myocarditis, our experience shows that when these ECG changes are detected in patients with multiple organ dysfunction and a history of mushroom consumption, mushroom poison‐induced toxic myocarditis should be considered.

## AUTHOR CONTRIBUTIONS

Liangqing Ge contributed to study conceptualization, writing—original draft, and final approval. Zuoan Qin contributed to investigation, writing—original draft, and data collection. Li Luo contributed to methodological analysis and conceptualization. All authors cared about patients and approved the final manuscript.

## FUNDING INFORMATION

This article is funded by the Technology Research and Program Clinical Medical Research Center (No.2021SK4042).

## CONFLICT OF INTEREST

The authors declare no competing interests related to this work.

## ETHICAL APPROVAL

The study was approved by the ethical committee of The First People's Hospital of Changde City followed by the ethical Declaration of Helsinki.

## Data Availability

Data sharing not applicable.
